# Bedside Ultrasound Measures of Muscle Mass and Frailty Measures in Community-Dwelling Older Adults

**DOI:** 10.1093/geroni/igaa057.861

**Published:** 2020-12-16

**Authors:** Kenneth Madden, Boris Feldman, Shane Arishenkoff, Graydon Meneilly

**Affiliations:** University of British Columbia, Vancouver, British Columbia, Canada

## Abstract

The age-associated loss of muscle mass and strength in older adults is called sarcopenia, and it is associated with increased rates of falls, fractures, hospitalizations and death. Sarcopenia is one of the most common physical etiologies for increased frailty in older adults, and some recent work has suggested the use of Point-of care ultrasound (PoCUS) measures as a potential measure of muscle mass. The objective of this study was to examine the association of PoCUS measures of muscle thickness (MT) with measures of frailty in community-dwelling older adults. We recruited 150 older adults (age >= 65; mean age 80.0±0.5 years, 66 women, 84 men) sequentially from 5 geriatric medicine clinics (Vancouver General Hospital). We measured lean muscle mass (LMM, by bioimpedance assay) and an ultrasonic measure of muscle quantity (MT, vastus medialis muscle thickness) in all subjects, as well as two outcome measures of frailty (FFI, Fried Frailty Index; RCFS, Rockwood Clinical Frailty Scale). In our models, MT showed an inverse correlation with the FFI (Standardized β=-0.2320±0.107, p=0.032) but no significant correlation with the RCFS (Standardized β = -0.025±0.086, p=0.776). LMM showed no significant association with either FFI (Standardized β=-0.232±0.120, p=0.055) or RCFS (Standardized β = -0.043±0.119, p=0.719). Our findings indicate that PoCUS measures show potential as a way to screen for physical manifestations of frailty and might be superior to other bedside methods such as bioimpedance assay. However, PoCUS measures of muscle thickness will likely miss patients showing frailty in the much broader context captured by the RCFS.

